# Binding Properties of Odorant-Binding Protein 4 of *Tirathaba rufivena* to *Areca catechu* Volatiles

**DOI:** 10.3390/plants11020167

**Published:** 2022-01-09

**Authors:** Xiang Zhou, Zheng Wang, Guangchao Cui, Zimeng Du, Yunlong Qian, Shumei Yang, Minghui Liu, Jixing Guo

**Affiliations:** Key Laboratory of Green Prevention and Control of Tropical Plant Diseases and Pests (Ministry of Education), College of Plant Protection, Hainan University, Haikou 570228, China; zhouxiangdc@aliyun.com (X.Z.); wangzheng56@hainanu.edu.cn (Z.W.); cuiguangchao@hainanu.edu.cn (G.C.); duzimeng@hainanu.edu.cn (Z.D.); hainanuqyl@hainanu.edu.cn (Y.Q.); yangshumei@hainanu.edu.cn (S.Y.); liuminghui@hainanu.edu.cn (M.L.)

**Keywords:** odorant-binding proteins, *Tirathaba rufivena*, binding ability, fluorescence competitive binding assays, molecular docking

## Abstract

Odorant-binding proteins (OBPs) play a key role in the olfactory system and are essential for mating and oviposition host selection. *Tirathaba rufivena*, a serious lepidopterous insect pest of the palm area in recent years, has threatened cultivations of *Areca catechu* in Hainan. Female-biased odorant-binding protein 4 of *T. rufivena* (TrufOBP4) expression was hypothesized to participate in the process of oviposition host recognition and localization. In this study, we cloned and analyzed the cDNA sequence of TrufOBP4. The predicted mature protein TrufOBP4 is a small, soluble, secretory protein and belongs to a classic OBP subfamily. Fluorescence binding assay results showed that TrufOBP4 had high binding abilities with the host plant volatiles, octyl methoxycinnamate, dibutyl phthalate, myristic acid and palmitic acid. These four components tend to dock in the same binding pocket based on the molecular docking result. The interactions and contributions of key amino acid residues were also characterized. This research provides evidence that TrufOBP4 might participate in the chemoreception of volatile compounds from inflorescences of *A. catechu* and can contribute to the integrated management of *T. rufivena*.

## 1. Introduction

The olfactory system of insects plays an important role in behaviors such as host seeking, mating, and oviposition [1]. Insects can acquire information from chemical odorants in the external environment and respond accordingly [2]. The process of odor molecular recognition in insects is a very complex chain reaction [3,4,5]. The water-soluble carriers are required to transport the lipophilic odorant molecule to receptive membranes. Odorant binding proteins (OBPs) are one of the major protein classes responsible for the binding and transport of water-insoluble compounds through the sensillar lymph [6,7]. OBPs are small and water-soluble proteins, and a typical OBP contains six conserved cysteine residues [8]. An inner hydrophobic pocket used for lipophilic ligand binding is established by the interconnection of disulfide bridges [9,10]. In addition, OBPs with more or less than six conserved cysteines have been identified and designated Plus-C OBPs and Minus-C OBPs, respectively [1,11].

The first OBP of lepidopteran insects was reported in the polyphemus moth *Antheraea Polyphemus* L. (Cramer) (Lepidoptera: Saturniidae) and found to bind sex pheromones [12]. Two specific subclasses, general odorant-binding proteins (GOBPs) and pheromone binding proteins (PBPs), were identified in lepidopteran species based on the distribution pattern in sensilla and similarities of amino acid sequences [13,14,15]. A large number of GOBPs and PBPs have been identified in the past decade [16,17,18]. OBPs possess high-specificity binding affinity with different plant volatiles and sex pheromones. Based on fluorescence competitive binding assays and computational molecular docking methods, a wide range of volatile binding capabilities have been reported [19]. However, it is still of great significance to characterize both the GOBPs and PBPs from different target insects chosen for study because the insect olfactory system is complex.

*Tirathaba rufivena* Walker (Lepidoptera: Pyralidae) is an important pest of areca palm, *Areca catechu* Linn. (Arecales, Arecaceae), in China [20]. This pest has seriously threatened areca nut cultivation in Hainan Province in recent years. Adult females usually lay eggs inside the gap at the base of the inflorescence before it unfolds. Newly hatched larvae penetrate the spathe and feed on the inflorescence. During the flowering and fruiting stages, the larvae can also eat petals and nuts, resulting in reduced yield [20]. Due to the invisibility of pests, the use of chemical pesticides cannot achieve good control effects, and the trees of *A. catechu* are usually tall, increasing the difficulty of pesticide application [21].

Odorant-binding proteins have been considered as pest control targets. Based on analyses of binding affinity, molecular docking and 3D crystal structures, a large number of new powerful semiochemicals have been identified, accelerating the discovery of active chemicals that could be used to manipulate insect behaviors for pest management [22]. Volatiles from areca inflorescence play roles in communication and guiding oviposition host localization [23,24,25]. The volatile components in areca inflorescence were determined and analyzed by GC-MS [26,27]. The combination of OBP from *T. rufivena* with semiochemicals is the key process by which *T. rufivena* finds a suitable host and finishes the reproduction process. Our previous study suggested that TrufOBP4 was highly expressed in female adults. It was hypothesized that TrufOBP4 has a possible functional role in the female-specific odor recognition process. To examine this hypothesis, we cloned the cDNA sequence of TrufOBP4; the binding activities of TrufOBP4 to host plant volatiles of *A. catechu* were characterized; and key amino acid residues that contributed to their binding interactions were identified. This study will provide evidence that TrufOBP4 might be involved in the chemoreception of host volatile compounds and can successfully contribute to the integrated management of *T. rufivena*.

## 2. Results

### 2.1. Sequence Analysis of TrufOBP4

**Figure 1:**
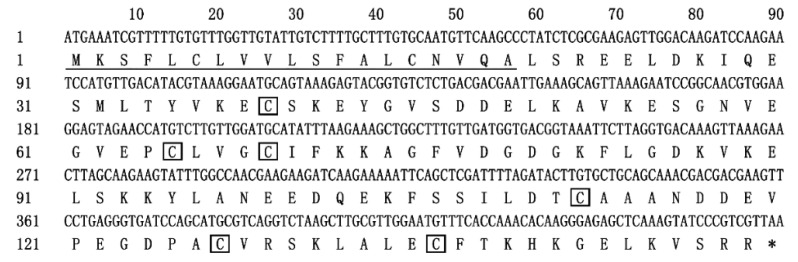
The cDNA and deduced amino acid sequences of TrufOBP4. The predicted signal peptide is indicated by underlining. The conserved Cys sites are indicated in black boxes. The translation-termination codon is marked by an asterisk *.

Based on the transcriptome data of *T. rufivena*, the cDNA sequence of TrufOBP4 was cloned and submitted to the NCBI GenBank database (Accession No: OK484430). The TrufOBP4 sequence contained an open reading frame (ORF) of 450 bp and encoded 149 amino acids with a 19 amino acid residue signal peptide at the N-terminus (Figure 1). The molecular mass and acidic isoelectric point of the predicted mature protein were 16.5 kDa and 4.98, respectively. The hydropathic nature of TrufOBP4 calculated and plotted for each residue showed that the grand average hydropathicity was −0.578 and that the four residue regions were hydrophobic. 

**Figure 2:**
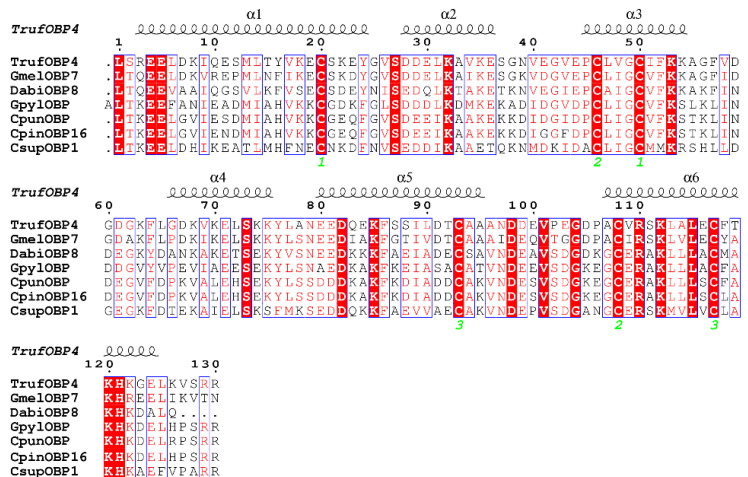
Alignment of TrufOBP4 with OBP genes from other Pyraloidae insect species. *Galleria mellonella* (Gmel), *Dioryctria abietella* (Dabi), *Glyphodes pyloalis* (Gpyl), *Conogethes punctiferalis* (Cpun), *Conogethes pinicolalis* (Cpin), and *Chilo suppressalis* (Csup). The bound cysteines that formed disulfide bridges are marked by green digits at the bottom of sequences.

We conducted an alignment of the amino acid sequence of TrufOBP4 with similar OBPs. The translated amino acid sequence of TrufOBP4 shows approximately 43.85–72.00% identity with the OBPs of other Pyraloidae insects. TrufOBP4 had the following conserved six-cysteine signature of OBPs: X_38_-Cys-X_25_-Cys-X_3_-Cys-X_42_-Cys-X_14_-Cys-X_8_-Cys-X_6_. Analysis with the Self-Optimized Prediction Method with Alignment (SOPMA) demonstrated that the α-helix was the main structure of TrufOBP4 (63.85%), in addition to random coils (25.38%), β turns (8.46%), and extended strands (2.31%) (Figure 2). No hydrophobic transmembrane helices were found in TrufOBP4 by ESPript3.0 [28]. 

**Figure 3:**
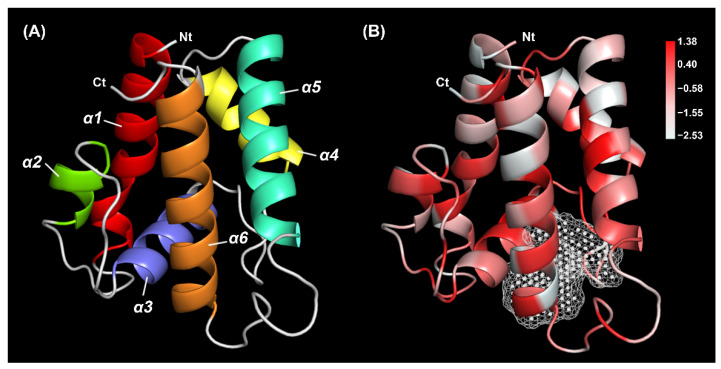
Three-dimensional structure model of TrufOBP4. (**A**) The 3D structures of TrufOBP4 with six helices labeled and colored by the rainbow. (**B**) The hydrophobic profiles and predicted binding pocket of TrufOBP4. The levels of hydrophobic value were illustrated by a gradient color scale, and the binding pocket was marked by white dots. Nt: N-terminus, Ct: C-terminus.

The 3D structure model was constructed using the crystal structures of *B. mori* pheromone binding protein (20.97% identity) as a template model. The quality and accuracy of the predicted model were evaluated. The Ramachandran plot showed 91.2% of residues in the favorable region, and 97.4% of all residues were in allowed regions (Figure S1A). The Verify 3D results showed that 90.63% of TrufOBP4 residues scored above 0.2 (Figure S1B). This protein had a secondary structure consisting of six α-helices, which are located between residues Arg22 and Tyr43 (α1), Asp47 and Glu55 (α2), Pro64 and Lys74 (α3), Gly85 and Tyr95 (α4), Glu99 and Ala114 (α5), and Ala126 and Glu143 (α6) (Figure 3A). The framework of helices was stabilized by three highly conserved internal disulfide bridges with six cysteine residues, Cys39-Cys69, Cys65-Cys136 and Cys112-Cys127. A pocket surrounded by hydrophobic residues was observed in the form of a tunnel occupying approximately 223 Å^3^ (Figure 3B).

### 2.2. Phylogenetic Tree Construction of TrufOBP4

**Figure 4:**
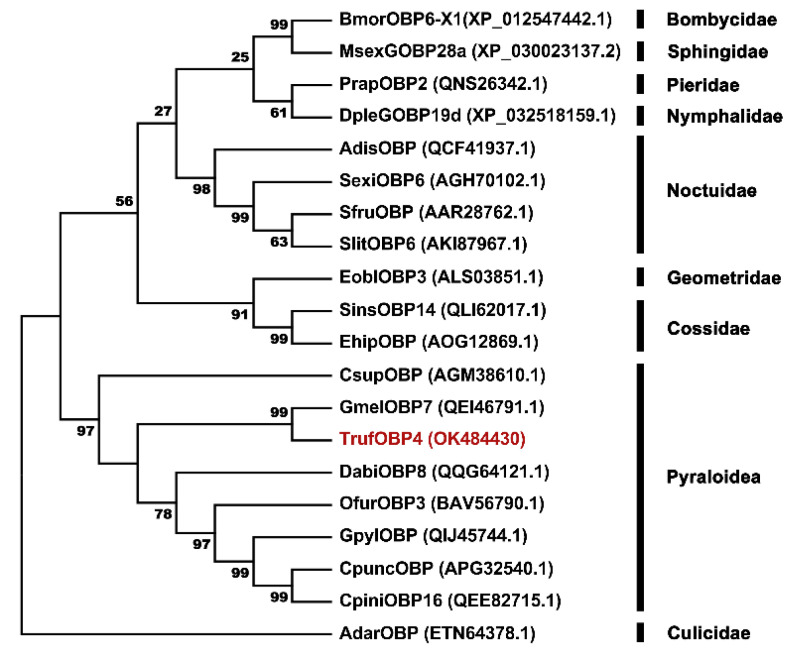
Phylogenetic tree of TrufOBP4 amino acid sequences with OBPs from other lepidopteran and dipteran insect species. The tree was constructed using the neighbor-joining method with a bootstrap of 1000 replicates. AdarOPB from *Anopheles darlingi* (Diptera: Culicidae) were used as the outgroup to root the tree. *Anopheles darlingi* (Adar), *Athetis dissimilis* (Adis), *Bombyx mori* (Bmor), *Conogethes pinicolalis* (Cpin), *Conogethes punctiferalis* (Cpun), *Chilo suppressalis* (Csup), *Dioryctria abietella* (Dabi), *Danaus plexippus* (Dple), *Ectropis obliqua* (Eobl), *Eogystia hippophaecolus* (Ehip), *Galleria mellonella* (Gmel), *Glyphodes pyloalis* (Gpyl), *Manduca sexta* (Msex), *Ostrinia furnacalis* (Ofur), *Pieris rapae* (Prap), *Spodoptera exigua* (Sexi), *Spodoptera frugiperda* (Sfru), *Streltzoviella insularis* (Sins), and *Spodoptera litura* (Slit).

A phylogenetic tree was constructed with TrufOBP4 and nineteen OBP sequences of other species of Lepidoptera to assess evolutionary relationships among the proteins. TrufOBP4 was clustered with GmelOBP7 (QEI46791.1), DabiOBP8 (QQG64121.1), OfurOBP3 (BAV56790.1), GpylOBP (QIJ45744.1), and CsupOBP (AGM38610.1) in the Pyraloidea superfamily, in which TrufOBP4 was close to the protein GmelOBP7 (QEI46791.1) with 72.00% similarity (Figure 4).

### 2.3. Expression and Purification of Recombinant TrufOBP4

**Figure 5:**
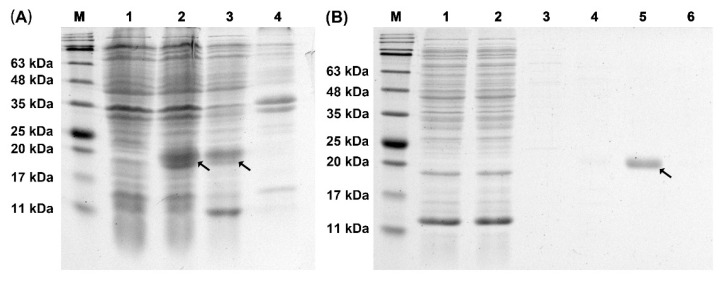
Expression and purification of recombinant protein TrufOBP4 analyzed by SDS-PAGE. (**A**) SDS-PAGE analysis of the expression of recombinant TrufOBP4. M: Protein molecular weight marker. 1: Protein sample without IPTG induction; 2: Protein sample with 0.5 mM IPTG induction. 3: The supernatant of protein sample with 0.5 mM IPTG induction after ultrasonic crushing. 4: The precipitation of protein sample with 0.5 mM IPTG induction after ultrasonic crushing. (**B**) SDS-PAGE analysis of the purification of recombinant TrufOBP4. M: Protein molecular weight marker. 1: Protein fluid after flowing through the column. 2–6: Protein samples were obtained with imidazole elution at different concentrations (0, 40, 100, 200 and 1000 mmol/L imidazole). The target protein was marked by an arrow.

The recombinant TrufOBP4 protein was successfully expressed as a soluble protein in the supernatant of *E. coli* BL21 (DE3) after induction with isopropyl β-D-1-thiogalactopyranoside (IPTG). After ultrasonication, the proteins were purified using Ni-NTA resin affinity chromatography. Then, the His-tag was removed by enterokinase. After confirmation by SDS–PAGE, the purified OBP proteins were further used to test the binding properties (Figure 5).

### 2.4. Fluorescence Competitive Binding Assays

**Figure 6:**
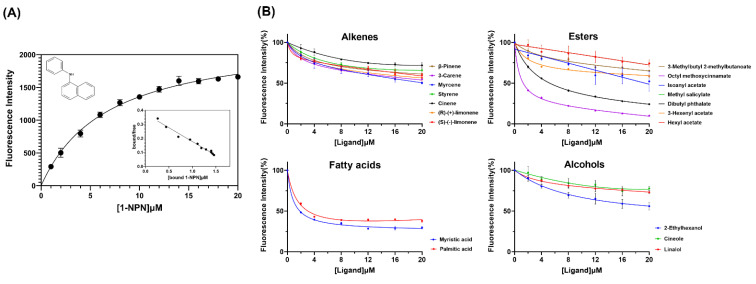
Competitive fluorescence ligand-binding assay of TrufOBP4 to volatiles. (**A**) Binding curve and relative Scatchard plot of TrufOBP4 and 1-NPN. (**B**) Competitive binding curves of TrufOBP4 with alkene, esters, fatty acids and alcohols.

Using competitive binding assays, we tested nineteen volatiles from areca inflorescences as competitors. The fluorescent reporter 1-phenyl-1-naphthylamine(1-NPN) was employed to test the binding affinity constants with TrufOBP4. Scatchard plots were analyzed to calculate the dissociation constant. The TrufOBP4 binds to 1-NPN with a K1-NPN of 6.79 μM (Figure 6). The K1-NPN value was then used to calculate the Ki values of nineteen volatiles with TrufOBP4. The competitive fluorescence binding curves showed that most of the ligands could reduce the relative fluorescence intensity of the TrufOBP4/1-NPN complex, indicating that TrufOBP4 could bind to all nineteen volatiles. The compounds octyl methoxycinnamate, dibutyl phthalate, myristic acid and palmitic acid had high binding affinities to TrufOBP6 with Ki values of 1.16, 4.61, 3.52 and 5.28 μM, respectively (Table 1).

### 2.5. Molecular Docking

**Figure 7:**
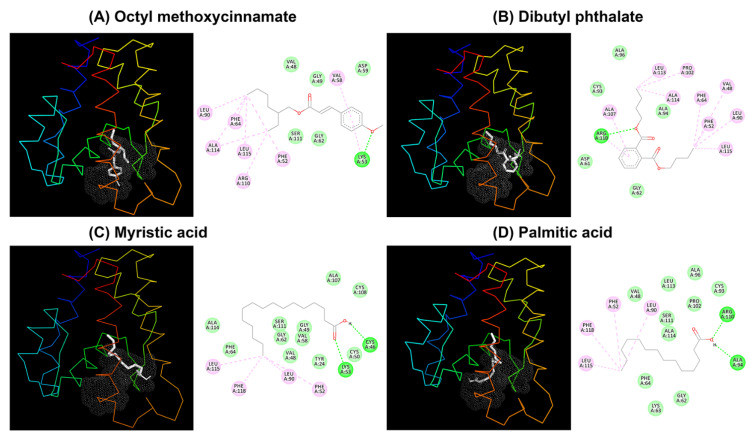
Molecular docking of TrufOBP4 with ligands. (**A**) 3D predicted interaction view of binding pocket (left) and 2D predicted interaction (right) of TrufOBP4 and octyl methoxycinnamate. (**B**) 3D predicted interaction view of binding pocket (left) and 2D predicted interaction (right) of TrufOBP4 and dibutyl phthalate. (**C**) 3D predicted interaction view of binding pocket (left) and 2D predicted interaction (right) of TrufOBP4 and myristic acid. (**D**) 3D predicted interaction view of binding pocket (left) and 2D predicted interaction (right) of TrufOBP4 and palmitic acid.

To further investigate the binding mode of TrufOBP4 with the chemicals and validate the results of the ligand-binding assay, the tested ligands with the highest binding ability (Ki below 10 µM) were chosen and docked against TrufOBP4. These four compounds, octyl methoxycinnamate, dibutyl phthalate, myristic acid and palmitic acid, exhibited good interactions against TrufOBP4 with binding energies of −5.24, −5.91, −5.69 and −5.11, respectively. All compounds tended to dock in the same binding pocket. Van der Waals interactions, a hydrogen bond (H-bond) and covalent interactions were found between TrufOBP4 and the compounds (Figure 7). 

Octyl methoxycinnamate showed van der Waals interactions toward Val48, Gly49, Asp59, Gly62 and Ser111, while it formed covalent interactions with Phe52, Val58, Phe64, Leu90, Arg110, Ala114 and Leu115. Only Lys53 formed an H-bond. Dibutyl phthalate bound against Asp61, Gly62, Cys93, Ala94 and Ala96 through van der Waals interactions and Val48, Phe52, Phe64, Leu90, Pro102, Ala107, Ala114, Leu113 and Leu115 through covalent interactions. Only Arg110 formed an H-bond. Myristic acid formed an H-bond with Cys46 and Lys53. Phe52, Leu90, Leu115 and Phe118 were involved in covalent bonding, while Tyr24, Val48, Gly49, Cys50, Val58, Gly62, Phe64, Ala107, Cys108, Ser111 and Ala114 were bound through van der Waals interactions. During palmitic acid and TrufOBP4 binding, Phe52, Leu90, Leu115 and Phe118 were involved in covalent bonding, while Val48, Gly62, Phe64, Cys93, Ala96, Pro102, Ser111, Leu113 and Ala114 were bound through van der Waals interactions, and Ala94 and Arg110 formed an H-bond. 

## 3. Discussion

The insect olfaction system allows insects to recognize and track volatile cues from host plants and mates, avoid toxic compounds and evade their predators [19]. The OBPs play an important role in olfactory sensation and are essential for binding odorant molecules and facilitating their transport through the aqueous neuronal environment [29]. It was suggested that TrufOBP4, one of the most abundant OBPs expressed in females, could participate in the female-specific olfactory recognition process. In this study, we cloned and analyzed the cDNA sequence of TrufOBP4. The predicted mature protein TrufOBP4 was a small, soluble, secretory protein with a molecular mass of 16.5 kDa and 19 amino acid residue signal peptides at the N-terminus. The hydropathic nature of TrufOBP4 was very similar to that of other insect OBPs. The sequences of TrufOBP4 displayed the conserved “OBP sequence motif of Lepidoptera”, and the conserved motif was C_1_-X_25-30_-C_2_-X_3_-C_3_-X_36–42_-C_4_-X_8–14_-C_5_-X_8_-C_6_ [30,31]. Three pairs of disulfide bridges were formed by six conserved cysteines, which demonstrated that TrufOBP4 could belong to classic OBP subfamilies. Amino acid sequence analysis of TrufOBP4 with similar OBPs indicated that TrufOBP4 shared high sequence identity with other Pyraloidae insects. TrufOBP4 was clustered with GmelOBP7 in the phylogenetic tree, which was consistent with the highest sequence similarity between them. This indicated that these homologous genes are evolutionarily conserved and play similar biological roles in olfaction. The 3D structure model showed that TrufOBP4 had a secondary structure consisting of six α-helices and internal cavity, which were stabilized by three highly conserved internal disulfide bridges.

Data from fluorescence binding assays showed that TrufOBP4 is specifically bound to octyl methoxycinnamate, dibutyl phthalate, myristic acid and palmitic acid with high binding affinities. The docking result also confirmed the interactions of these four compounds with TrufOBP4. The binding capacity of TrufOBP4 with host plant volatiles was attributed to van der Waals interactions, hydrogen bonds, and covalent interactions (Pi alkyls). Hydrophobic residues, such as V48, F52, G62, F64, L90, A114, and L115, which were highly overlapped, were conserved, contributing to the interaction with odors rather than hydrophilic residues. Previous studies in *Apolygus lucorum* (Hemiptera: Miridae) also showed that mutants of hydrophobic residues decreased or completely abolished binding affinities to ligands. These amino acid residues play vital roles in the formation of the interaction between a protein and hydrophobic ligand. The specific role of these residues needs further study through a site-directed mutagenesis technique [12,29,32,33,34].

The high binding affinities of octyl methoxycinnamate, dibutyl phthalate, myristic acid and palmitic acid to the female-biased expressed OBPs of adult *T. rufivena* suggested an essential role for these odor molecules in the chemoreception of volatiles from the host plant *A. catechu.* Dibutyl phthalate was reported to be a common component of many plant volatiles [35]. Many studies have demonstrated that dibutyl phthalate can influence the behavior of insects [24,25,36]. For example, this compound had an attractive effect on *Holotrichia oblita* Faldermann (Coleoptera: Scarabaeidae) but was repellent to *Rhyzopertha dominica* (Coleopera: Bostrichidae) and *Tribolium castaneum* (Coleoptera: Tenebrionidae) [35]. In addition, a study indicated that mating rates were significantly increased by the stimulation of *Hyphantria cunea* (Lepidoptera: Arctiidae) with dibutyl phthalate [24]. The OBPs of many insect species were also reported to have a high binding affinity to dibutyl phthalate. Some studies in lepidopteran demonstrated that AipsGOBP2 of *Agrotis ipsilon* (Lepidoptera: Noctuidae) and EoblGOBP2 of *Ectropis obliqua* (Lepidoptera: Geometridae) show an outstanding binding affinity to dibutyl phthalate [36,37]. In addition, the high binding affinities of OBPs with dibutyl phthalate were observed in other species [25]; for instance, the ability to perceive dibutyl phthalate of *H. oblita* females decreased once HoblOBP7 expression was knocked down [25,36]. Therefore, we propose that dibutyl phthalate can also influence the behavior of *T. rufivena*.

Myristic acid and palmitic acid were also proven to be among the best ligands for TrufOBP4. Some researchers have reported that OBP has a high binding ability with fatty acids [38]. The MbraPBP1 of *Mamestra brassicae* (Lepidoptera: Noctuidae) and LsatOBP1 of *Liriomyza sativae* Blanchard (Diptera: Agromyzidae) bind well to palmitic acid [12,39]. Fatty acids are not only basic components of insects and plants but also play important physiological roles in insects. The PregOBP56a, which showed a high binding affinity with palmitic, stearic, oleic, and linoleic acids, was proposed to solubilize these fatty acids and deliver them to the midgut in the reproductive process [38]. However, the olfactory peripheral coding mechanism of myristic acid and palmitic acid and its function in the physiological process still need further study.

## 4. Materials and Methods

### 4.1. Insect Rearing

Larvae of *T. rufivena* were collected from the host plant areca palms in Qionghai County (19.23° N, 110.47° E), Hainan province, China. The larvae were reared in the laboratory under environment conditions at 28 ± 1 °C, with a relative humidity of 80 ± 10% and a photoperiod of 14:10 (L:D) with an artificial diet.

### 4.2. RNA Extraction and cDNA Synthesis

Antennas from ten female adults (first generation) were collected and immediately frozen in liquid nitrogen. Total RNA was extracted using TRIzol reagent (Invitrogen, Carlsbad, USA). RNA integrity was determined by 1% agarose gels. RNA quantity and purification were checked using a NanoPhotometer Spectrophotometer (Implen, CA, USA). First-strand cDNA was synthesized by a HiScript^®^II Reverse Transcriptase synthesis kit (Vazyme, Nanjing, China) according to the manufacturer’s instructions.

### 4.3. Cloning and Sequencing Analysis 

Specific primers were designed and synthesized by Sangon Biotech Company (Shanghai, China) based on the nucleotide sequence of TrufOBP4. The sense primer was 5-TGTGACGCTTTACACTAA-3, and the antisense primer was 5-AGTCGTCTAATCAGGAAA-3. PCR studies were performed using Phanta Max Super-Fidelity DNA Polymerase (Vazyme, Jiangsu, China). The amplification of the TrufOBP4 cDNA fragment was performed on a Veriti Thermal Cycler (AB, CA, USA) with the following PCR procedure: 94 °C for 5 min; 30 cycles at 94 °C for 30 s, at 60 °C for 30 s, and at 72 °C for 30 s; and 72 °C for 10 min. The colonies were sequenced by Sangon Biotech Company (Shanghai, China). The cDNA sequence and deduced amino acid sequence of TrufOBP4 were analyzed using DNAMAN (Lynnon Biosoft, CA, USA). The putative N-terminal signal peptides were predicted using the SignalP V4.1 program (https://services.healthtech.dtu.dk/service.php?SignalP-4.1 (accessed on 10 January 2021)). The chemical and physical properties, secondary structure and hydrophobicity scales of TrufOBP4 were predicted using the online program tools ProtParam (https://web.expasy.org/protparam/ (accessed on 10 January 2021)), SOPMA (https://npsa-prabi.ibcp.fr/cgi-bin/ (accessed on 10 January 2021)), ProtScale (https://web.expasy.org/protscale/ (accessed on 15 January 2021)) and ESPript3.0 software [28]. The larger the hydrophobic value, the stronger the hydrophobicity of this amino acid [40]. ClustalW and MEGA 5.2 were employed for multiple alignments and the phylogenetic tree construction of TrufOBP4 with similar OBPs of other insect species using the neighbor-joining method with a No. of differences model and a pairwise deletion of gaps.

### 4.4. Expression and Purification of Recombinant TrufOBP4 

The cDNA sequence of TrufOBP4-removed signal peptides was amplified using designed primers. The enzymatic digestion sites were designated EcoRI and NotI. The sense primer was 5-CGGAATTCCTATCTCGCGAAGAGTTGG-3 (the EcoRI restriction site is underlined), and the antisense primer was 5-AAATATGCGGCCGCACGACGGGATACTTTGAGCT-3 (the NotI restriction site is underlined). The purified PCR product and pET-32a (+) vector (Solarbio, Beijing, China) were ligated and transformed into *E. coli* BL21 (DE3) cells (AxyGen, Shanghai, China). Cells of *E. coli* were cultured until the OD600 value reached 0.6, while β-D-1-thiogalactopyranoside (IPTG) was added to the LB medium at a final concentration of 0.5 mM. The bacterial cultures were shaken continuously at 200 rpm at 30 °C for 6 h to induce recombinant TrufOBP4 protein. The suspension was crushed by sonication and then separated into supernatant and sediment by centrifugation (6000 rpm, 10 min, 4 °C). Then, pET-32a (+)-TrufOBP4 was purified and desalted using Ni Sepharose 6FF (Solarbio, Beijing, China) and Amicon^®^ Ultra-4 3K centrifugal filters (Merck Millipore, Darmstadt, Germany), respectively. The size and purity of the recombinant protein were analyzed with 12% SDS–PAGE.

### 4.5. Fluorescence Competitive Binding Assays

The binding ability of purified recombinant OBPs was evaluated with an F-7000 fluorescence spectrometer (Hitachi, Tokyo, Japan) [41,42]. The fluorescence spectra were recorded between 360 and 650 nm. *N*-Phenyl-1-naphthylamine (1-NPN) was selected as the fluorescent reporter and dissolved in chromatographic methanol at a concentration of 1 mM. The purified TrufOBP4 was dissolved in 50 mM Tris-HCl (pH 7.4) at a final concentration of 2 μM. The data for the 1-NPN-TrufOBP4 complex formation were obtained by titration of 2 μM protein with increasing concentrations (2 to 20 μM) of 1-NPN. The dissociation constant of 1-NPN (K_1-NPN_) was calculated using Prism 8 (GraphPad Software, San Diego, USA). To evaluate the binding ability of TrufOBP4 with volatiles, 1-NPN was replaced with different odor compounds from the TrufOBP4/1-NPN complex. The odor ligands were dissolved in chromatographic methanol at a concentration of 1 mM and added as aliquots to the protein solution. The spectral results were obtained, and the dissociation constants of the competitors were calculated with the formula: Ki = IC50/(1 + [1-NPN]/K_1-NPN_), where 1-NPN represents the free concentration of 1-NPN, and IC50 represents the ligand concentration displacing 50% of the fluorescent reporter [43].

### 4.6. Structure Modelling and Molecular Docking

The predicted three-dimensional structure (3D) of TrufOBP4 was modelled using the SWISS-MODEL prediction algorithm (https://swissmodel.expasy.org/ (accessed on 5 April 2021)) with the crystal structure of BmorPBP (PDB: 2p70.1. A) selected as the template [44]. The three-dimensional (3D) conformer structures of candidate volatiles were downloaded from the chemical compound databases ZINC (https://zinc.docking.org/ (accessed on 5 March 2021)) and PubChem (https://pubchem.ncbi.nlm.nih.gov/ (accessed on 5 March 2021)). The 3D model quality assessment was performed using SAVES v6.0 (https://saves.mbi.ucla.edu (accessed on 9 April 2021)). The POCASA 1.1 program (https://g6altair.sci.hokudai.ac.jp/g6/service/pocasa/ (accessed on 5 April 2021)) was used to make a pocket prediction. AutoDock (Molecular Graphics Laboratory, La Jolla, CA, USA) was used to find the potential binding mode between TrufOBP4 and ligands. The binding affinity score was calculated based on the potential energy changes around the binding pocket during the protein–ligand interaction. A lower score corresponded to a stronger binding ability. Visual structure analysis was carried out by PYMOL Viewer (http://www.pymol.org/ (accessed on 20 April 2021)) and Discovery Studio visualizer (BIOVIA, San Diego, CA, USA).

## 5. Conclusions

In this study, the cDNA sequence of TrufOBP4, a female-biased expressed OBP, was cloned and analyzed. The TrufOBP4 showed high binding abilities with the host plant volatiles, octyl methoxycinnamate, dibutyl phthalate, myristic acid and palmitic acid based on fluorescence binding assays and molecular docking. The interactions and contributions of key amino acid residues were also characterized. This study provides evidence that TrufOBP4 might be involved in the chemoreception of host volatile compounds, and that a potential ecological-based trapping method can be developed by utilizing these common volatiles.

## Figures and Tables

**Table 1:** Binding affinities of different ligands to TrufOBP4.

No.	Ligands	Formula	CAS No#	IC50 (μM)	Ki (μM)
1	(R)-(+)-limonene	C_10_H_16_	5989-27-5	25.32	22.07
2	(S)-(−)-Limonene	C_10_H_16_	5989-54-8	33.36	29.08
3	2-Ethylhexanol	C_8_H_18_O	104-76-7	24.08	20.99
4	Octyl methoxycinnamate	C_18_H_26_O_3_	83834-59-7	1.33	1.16
5	3-Carene	C_10_H_16_	13466-78-9	22.41	19.53
6	Methyl salicylate	C_8_H_8_O_3_	119-36-8	>50	>50
7	Cinene	C_10_H_16_	138-86-3	>50	>50
8	Cineole	C_10_H_18_O	470-82-6	>50	>50
9	3-Hexenyl acetate	C_8_H_14_O_2_	3681-71-8	46.59	40.61
10	Dibutyl phthalate	C_16_H_22_O_4_	84-74-2	5.29	4.61
11	Hexyl acetate	C_8_H_16_O_2_	142-92-7	>50	>50
12	Isoanyl acetate	C_7_H_14_O_2_	123-92-2	24.11	21.02
13	3-Methylbutyl 2-methylbutanoate	C_10_H_20_O_2_	27625-35-0	>50	45.83
14	Linalol	C_10_H_18_O	78-70-6	>50	>50
15	Myrcene	C_10_H_16_	123-35-3	19.80	17.26
16	Myristic acid	C_14_H_28_O_2_	544-63-8	4.04	3.52
17	Palmitic acid	C_16_H_32_O_2_	57-10-3	6.06	5.28
18	Styrene	C_8_H_8_	100-42-5	41.50	36.17
19	β-Pinene	C_10_H_16_	18172-67-3	33.29	29.02

Note: “>50” for the IC50 and Ki means that IC50 or Ki cannot be accurately calculated with the ligand concentration range tested in the assay.

## Data Availability

All data included in the main text and Supplementary Materials.

## Supplementary Materials

The following are available online at https://www.mdpi.com/article/10.3390/plants11020167/s1, Figure S1: The model quality of TrufOBP4 evaluated by Procheck and Verify_3D.
