# Supplementary figures and images for: Binding Properties of Odorant-Binding Protein 4 of Tirathaba rufivena to Areca catechu Volatiles
Source: Plants (Basel). 2022 Jan 9;11(2):167. doi: 10.3390/plants11020167 (PMC8779631; doi:10.3390/plants11020167)

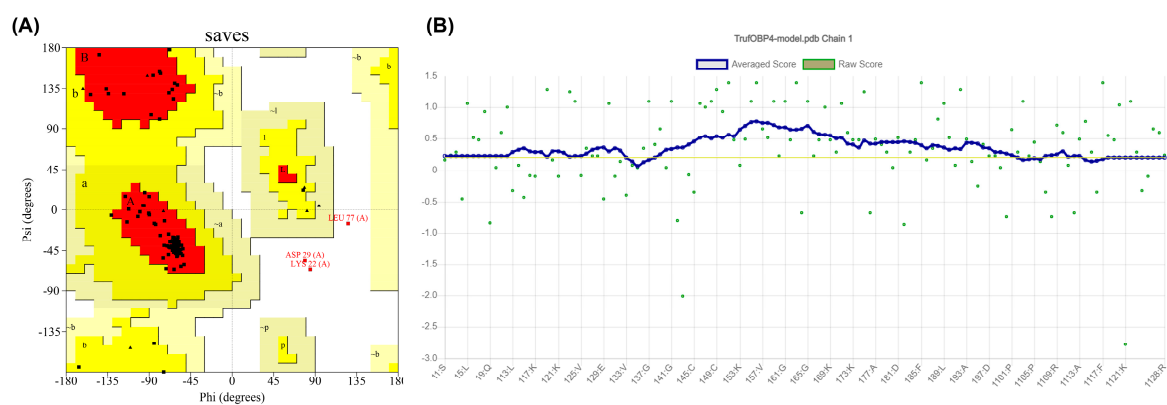

**Figure S1.** The model quality of TrufOBP4 evaluated by Procheck and Verify\_3D.

Supplement: Supplementary file 1 [file plants-11-00167-s001.zip › plants-1489044-supplementary.pdf]
